# Palladium(II)-catalyzed Heck reaction of aryl halides and arylboronic acids with olefins under mild conditions

**DOI:** 10.3762/bjoc.9.180

**Published:** 2013-08-05

**Authors:** Tanveer Mahamadali Shaikh, Fung-E Hong

**Affiliations:** 1 Department of Chemistry, National Chung Hsing University, 250 Kuo-Kuang Road, Taichung, Taiwan (R.O.C.)

**Keywords:** aryl halides, Heck reaction, olefins, palladium-complex, phosphine

## Abstract

A series of general and selective Pd(II)-catalyzed Heck reactions were investigated under mild reaction conditions. The first protocol has been developed employing an imidazole-based secondary phosphine oxide (SPO) ligated palladium complex (**6**) as a precatalyst. The catalytic coupling of aryl halides and olefins led to the formation of the corresponding coupled products in excellent yields. A variety of substrates, both electron-rich and electron-poor olefins, were converted smoothly to the targeted products in high yields. Compared with the existing approaches employing SPO–Pd complexes in a Heck reaction, the current strategy features mild reaction conditions and broad substrate scope. Furthermore, we described the coupling of arylboronic acids with olefins, which were catalyzed by Pd(OAc)_2_ and employed *N*-bromosuccinimide as an additive under ambient conditions. The resulted biaryls have been obtained in moderate to good yields.

## Introduction

Substituted olefins are important structural motifs in natural products, pharmaceuticals, bioactive compounds and organic materials [1–2]. Olefins such as stilbene derivatives normally show antitumor [3], antiinflammatory [4], neuroprotective [5], and cardioprotective [6] properties. Due to its importance in the synthesis of leading molecules, a variety of preparative methodologies have been developed. Particularly, the Heck reaction is one of the most chosen methods in the synthesis of aryl-substituted olefins [7–9]. Aryl halides or arylboronic acids are among the most commonly employed arylpalladium precursors in the Heck reaction.

In the early 1970s, Mizoroki [10] and Heck [11] developed a palladium(0)-catalyzed cross-coupling of olefins with organic halides. Later, several other catalytic protocols were used with variations in their coupling procedures by changing metal sources, ligands, additives or substrates [12–16]. The class of phosphine-ligated palladium complexes [17–21] represents the most frequently employed precatalysts to achieve high reactivities and selectivities for such reactions. However, such trisubstituted phosphines in the palladium complexes are often air-sensitive in nature and easily oxidized [22–23]. Therefore, a new class of secondary phosphine oxide ligands (SPO) has been explored for these ligand-assisted palladium-catalyzed cross-coupling reactions [24–27]. This type of SPO ligand is stable towards air and moisture and convenient to handle compared to the conventional trisubstituted phosphine ligands. Despite this advantage, the potential of these ligands has not been fully realized in Heck arylation reactions. Up to now, only a few examples of utilizing SPO-ligated palladium complexes in oxidative Heck reactions have been demonstrated [28–31]. Previously, we also reported the synthesis of cobalt-containing SPO ligands and their palladium complex. This was successfully applied as a catalytic precursor in oxidative Heck reactions [32]. However, these reactions were carried out at high temperatures with limited substrate scope. Therefore, the development of an alternative general and mild procedure employing a stable and inexpensive ligand is still in great demand.

Furthermore, the application of palladium complexes in the oxidative coupling of organo-boron compounds with olefins has attracted much attention in recent years [33–38]. Various catalytic systems have been developed by Jung [39] and Larhed et al. [40–43] by employing diverse variations in oxidants, ligands or palladium complexes [44–47]. Nowadays, several competent methods are also known to achieve this transformation with different reaction conditions employing base-free, ligand-free conditions or by using conventional oxidants such as oxygen, benzoquinone, Cu salts, etc. [48–53].

**Scheme 1:**
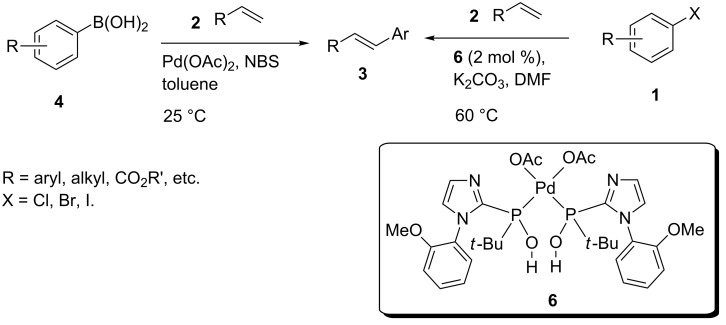
Heck reaction of olefins with aryl halides and arylboronic acids.

In this article we report two new protocols for Heck cross-coupling reactions: (i) a stable SPO ligated palladium complex **6** catalyzed cross-coupling of aryl halides **1** with olefins **2** at 60 °C; and (ii) Pd(OAc)_2_ catalyzed arylation of arylboronic acids **4** with olefins at 25 °C (Scheme 1).

## Results and Discussion

### Heck reaction of aryl halides with olefins

**Scheme 2:**
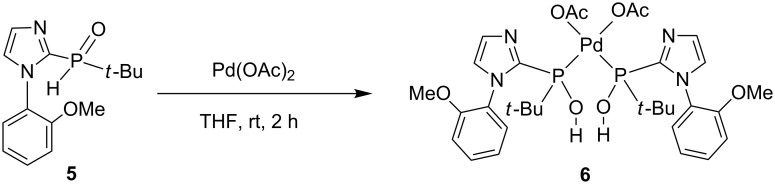
Synthesis of imidazole-based SPO–Pd complex **6**.

In the presence of solvents, secondary phosphine oxide (RR'P(O)H) might undergo tautomerization, which generates a less stable phosphinous acid (RR'POH) species. Subsequently, its coordination to the metal center through the phosphorus atom forms a phosphinous acid–metal complex [54–56]. Thus, the resulting transition-metal complex might function as an active catalyst in various C–C-bond-forming reactions. Ackermann et al. reported the synthesis of stable *N*-aryl-substituted pyrrole and indole-derived SPO-preligands, which were utilized in Kumada–Corriu cross-coupling reactions [57]. Recently, we reported the synthesis and characterization of imidazole-based secondary phosphine oxide ligand **5** and its application in C–C-bond-forming reactions (Scheme 2) [58]. Furthermore, the application of complex **6** in cross-coupling reactions has been carefully studied. We found that complex **6** is an active catalyst for the Heck reaction of aryl halides with olefins under mild conditions.

To optimize the reaction conditions, a series of reactions under various combinations of bases, solvents and temperatures, employing complex **6** as precatalyst, was pursued. Bromobenzene (**1a**) and styrene (**2a**) were chosen as the model substrates in this coupling reaction and the results are presented in Table 1.

**Table 1:** Palladium complex (**6**) catalyzed Heck reaction of bromobenzene and styrene: Optimization of reaction conditions.^a^

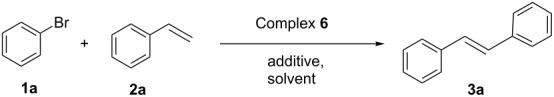					

Entry	Complex **6** (mol %)	Base (equiv)	Solvent	Temp (°C)	Yield (%)^b^

1	1	NaOH (1)	DMSO	rt	–
2	2	NaOAc (1)	DMSO	rt	–
3	2	Et_3_N (2)	DMSO	40	–
4	1	K_2_CO_3_ (1)	toluene	rt	–
5	1	K_2_CO_3_ (1)	CH_3_CN	rt	–^c^
6	1	K_3_PO_4_ (1)	THF	40	17
7	1	K_3_PO_4_ (1)	THF	60	24
8	1	K_2_CO_3_ (1)	THF	60	46
9	1	K_2_CO_3_ (1)	DMF	100	65
10	2	K_2_CO_3_ (2)	DMF	100	84
11	2	K_2_CO_3_ (2)	DMF	80	92
12	2	K_2_CO_3_ (2)	DMF	50	73
13	2	K_2_CO_3_ (1)	DMF	60	89
14	2	K_2_CO_3_ (2)	DMF	60	96
15	2	K_2_CO_3_ (2)	no solv.	60	82

^a^Reaction conditions: styrene (1.0 mmol), bromobenzene (1.0 mmol), base, solvent (1 mL), stirred for 12 h. ^b^Isolated yield. ^c^Reaction mixture was stirred for 24 h.

Initially, the coupling was carried out by using 1 mol % loading of Pd-complex **6** as a precatalyst, with styrene (**2a**, 1 mmol), and bromobenzene (**1a**, 1 mmol) in DMSO (2 mL), and at ambient temperature in the presence of NaOH (1 equiv, Table 1, entry 1). The reaction did not give the coupled product **3a**. Moreover, the use of other bases such as NaOAc, Et_3_N and K_2_CO_3_ in the presence of the solvents, DMSO, toluene or acetonitrile were not useful and no coupled product was observed. Interestingly, the reaction showed little progress in the presence of K_3_PO_4_ and tetrahydrofuran at 40 °C to obtain **3a** in 17% yield (Table 1, entry 6). The yield was slightly improved when the reaction was heated at 60 °C (Table 1, entry 7). When K_2_CO_3_ (1.0 equiv) in THF was employed under similar reaction conditions, the yield of *trans*-stilbene was improved to 46% (Table 1, entry 8). Once K_2_CO_3_ had been selected as the most effective base, the next step involved the enhancement of the product yield. The combination of K_2_CO_3_ (2 equiv) and DMF (2 mL) resulted in the formation of 84% of **3a** at 100 °C (Table 1, entry 10). A further increase in the reaction temperature would lead to decomposition of the palladium complex, which was formed in situ, thus lowered the yield of the product. Therefore, the loading of the precatalyst **6** was increased to 2 mol % and resulted in the formation of *trans*-stilbene in 92% yield at 80 °C (Table 1, entry 11). Synthetically, it is important to carry out reactions under mild reaction conditions. Nevertheless, low yield (73%) of the product was obtained by reducing the reaction temperature to 50 °C. Thus, a substrate survey was conducted at 60 °C. The optimized reaction conditions were found to be the use of styrene (**2a**, 1 mmol), bromobenzene (**1a**, 1 mmol), K_2_CO_3_ (2 mmol), and precatalyst **6** (2 mol %) with heating at 60 °C in DMF (1 mL, Table 1, entry 14). It is worthy of noting that the coupling reaction was also performed in the absence of solvent, which gave 82% yield (Table 1, entry 15) of the coupled product.

A wide range of olefins with different and diversely substituted aryl bromides were subjected to cross-coupling to produce the corresponding 1,2-disubstituted olefins. The results are summarized in Table 2.

**Table 2:** Heck reaction of olefins and aryl halides: Scope of substrate.^a^

Entry	Olefin (**2**)	Aryl halide (**1**)	Product (**3**)	Yield (%)^b,c^

1	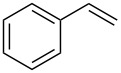 **2a**	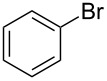 **1a**	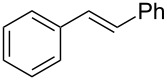 **3a**	96
2	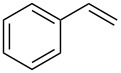 **2a**	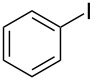 **1b**	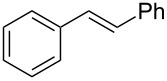 **3a**	98
3	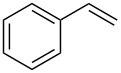 **2a**	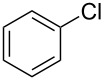 **1c**	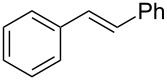 **3a**	62
4	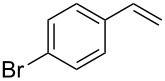 **2b**	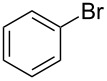 **1a**	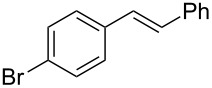 **3b**	90
5	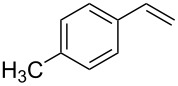 **2c**	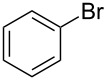 **1a**	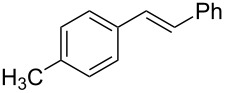 **3c**	92
6	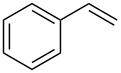 **2a**	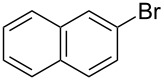 **1d**	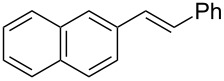 **3d**	88
7	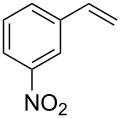 **2d**	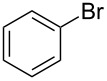 **1a**	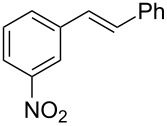 **3e**	90
8	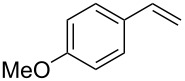 **2e**	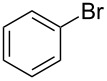 **1a**	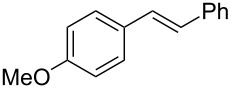 **3f**	95 (35)^d^
9	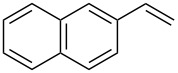 **2f**	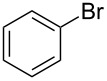 **1a**	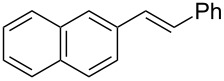 **3d**	91
10	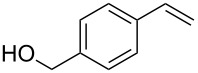 **2g**	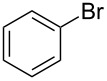 **1a**	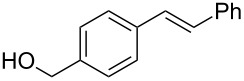 **3g**	80
11	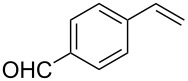 **2h**	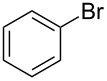 **1a**	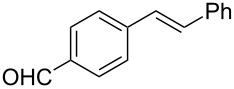 **3h**	85
12	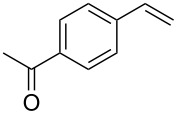 **2i**	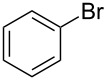 **1a**	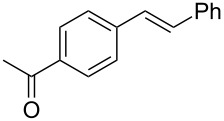 **3i**	87
13	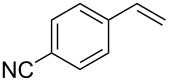 **2j**	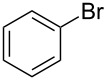 **1a**	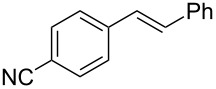 **3j**	90
14	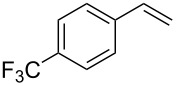 **2k**	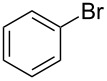 **1a**	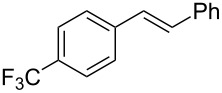 **3k**	89
15	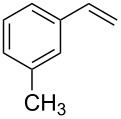 **2l**	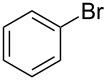 **1a**	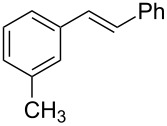 **3l**	88
16	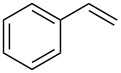 **2a**	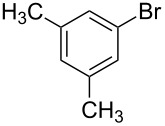 **1e**	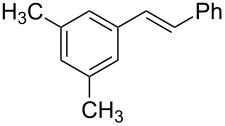 **3m**	90
17	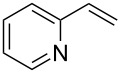 **2m**	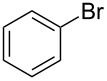 **1a**	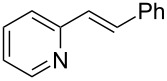 **3n**	78
18	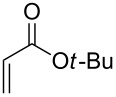 **2n**	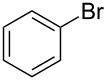 **1a**	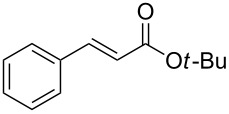 **3o**	95
19	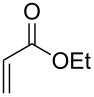 **2o**	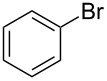 **1a**	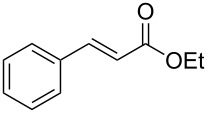 **3p**	90
20	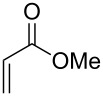 **2p**	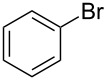 **1a**	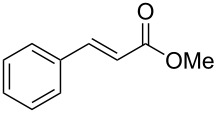 **3q**	92

^a^Reaction conditions: olefin (1.0 mmol), aryl halide (1.0 mmol), Pd-complex **6** (2.0 mol %), K_2_CO_3_ (2.0 mmol), DMF (1 mL), 60 °C, 12 h. ^b^Isolated yield. ^c^Products were characterized by ^1^H, ^13^C NMR and GC–MS. ^d^The yield corresponds to employing 4-chloro anisole as the aryl halide source.

Both aryl bromide and aryl iodide performed well (Table 2, entries 1 and 2) under these conditions. However, the aryl chloride was found to be less reactive giving the corresponding product **3a** in 62% yield (Table 2, entry 3). The oxidative coupling was found to be selective in the case of 4-bromostyrene (**2b**), which gives 90% yield of 4-bromo *trans*-stilbene (**3b**) without the observation of any side product (Table 2, entry 4). The presence of either an electron-withdrawing or electron-donating group on the aromatic ring of olefin did not affect the reactivity and yield of product. The reactions led to the formation of excellent yields of the corresponding products **3e** and **3f** in 90% and 95% yields, respectively (Table 2, entries 7 and 8). As known, aromatic rings having substituents such as, -CH_2_OH, -CHO, -COCH_3_ -CN and -CF_3_ are rather useful in organic synthesis. However, in earlier reported oxidative coupling conditions these functional groups were not compatible and gave low yields of products. Therefore, these highly modifiable groups were screened under these catalytic conditions. Thus, 4-vinylbenzyl alcohol (**2g**), 4-vinyl benzaldehyde (**2h**), 4-vinylacetophenone (**2i**), 4-cyanostyrene (**2j**) and 4-trifluoromethylstyrene (**2k**) were smoothly converted to their corresponding coupled products **3g**–**3k** in excellent yields (Table 2, entries 10–14). The selectivities and yields of the coupled products were excellent regardless of *ortho*-, *meta*-, or *para*-substitution patterns on either styrenes or aryl halides under these catalytic conditions. For example, the coupling of substituted methylstyrenes (Table 2, entry 15) or alkyl-substituted aryl halides (Table 2, entry 16) gave 88–90% isolated yields of **3l** and **3m**. To investigate whether the reaction was compatible with a heteroaryl olefin, 2-vinylpyridine (**2m**) was subjected to this reaction. It produced the corresponding coupled product **3n** in 78% yield (Table 2, entry 17). Furthermore, using these optimized conditions, bromobenzene (**1a**) was examined with different vinyl esters to determine the scope of this procedure. The results are given in Table 2, entries 18–20. Notably, the performances were in agreement with the previous expectations and yields are excellent in the preparation of α,β–unsaturated esters. The corresponding α,β-unsaturated esters **3o**–**3q** were obtained in 90–95% yields, respectively.

### Heck reaction of arylboronic acids with olefins

The phosphine- and base-free coupling of arylboronic acids with olefins under mild reaction conditions were studied as well to broaden the scope of cross-coupling reactions. To search for the optimized reaction conditions, phenylboronic acid (**4a**) and styrene (**2a**) were chosen as the model substrates and Pd(OAc)_2_ was employed as the catalyst. Various reaction conditions were tested and the results are presented in Table 3. Initially, a Pd(OAc)_2_ catalyzed Heck reaction was performed employing polar sovents, dimethylacetamide (DMAc) and DMF, at 25 °C in the presence of 0.5 equiv of *N*-bromosuccinimide (NBS). This resulted in the formation of *trans*-stilbene (**3a**) in 52% and 40% yield, respectively (Table 3, entries 1 and 2). However, the same reaction under the control conditions (i.e., in the absence of NBS) resulted in production of a trace amount of the coupled product **3a** (Table 3, entry 3). When the coupling reaction was carried out at 90 °C in DMAc solvent, the yield of **3a** decreased, due to the formation of side product, such as bromobenzene, from the corresponding phenylboronic acid (Table 3, entry 4). Therefore, it is believed that NBS plays an important role in this catalytic reaction. Furthermore, we focused our attention to other solvents such as MeOH, CH_2_Cl_2_, CH_3_CN, Me_2_O, *t*-Bu_2_O, THF, DMSO and 1,4-dioxane, which resulted in low yields of arylated product. Subsequently, the reaction was subjected to the apolar solvent toluene. The expected product *trans*-stilbene (**3a**) was obtained in 68% yield at 25 °C for 18 h (Table 3, entry 5). The yield of the desired product did not improve even when the reaction was stirred for 24 h (Table 3, entry 6). On the other hand, lowering the additive (NBS) to 10 mol % did not show any improvement to the formation of *trans*-stilbene (**3a**) (Table 3, entries 7 and 8). A sharp decline in the formation of *trans*-stilbene (**3a**) (Table 3, entry 9) was observed on increasing the quantity of NBS to a stoichiometric amount (1.0 equiv). This was probably due to the formation of other competitive side product(s). Interestingly, the coupled product was obtained with improved yield of 76% by using 30 mol % NBS (Table 3, entry 10). Next, we turned our attention to the improvement of the yields of *trans*-stilbene by adjusting other reaction parameters. Thus, the addition of K_2_CO_3_ as base along with NBS under similarly performed reaction conditions led to no formation of the targeted product. The addition of molecular sieves was not a good choice either [59]. The other additives such as LiBr and CuBr were also examined. Still, no coupled product was obtained in the presence of LiBr (30 mol %, Table 3, entry 13). On the other hand, the employment of CuBr (30 mol %) with the presence of Pd(OAc)_2_ resulted in a 42% yield of *trans*-stilbene (**3a**) (Table 3, entry 14). Thus, the optimized reaction conditions for the Heck reaction here is the use of arylboronic acid (1 mmol), olefin (1 mmol), Pd(OAc)_2_ (5 mol %), NBS (30 mol %), toluene (1 mL) at 25 °C under stirring for 12 h.

**Table 3:** Heck reaction of phenylboronic acid and styrene: Optimization of the reaction conditions.^a^

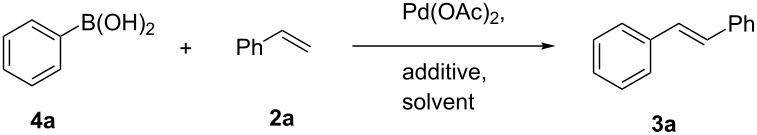					

Entry	Additive (equiv)	Solvent	Time (h)	Temp. (°C)	Yield (%)^b,c^

1	NBS (0.5)	DMAc	18	25	52
2	NBS (0.5)	DMF	18	25	40
3	–	DMAc	24	25	trace
4	NBS (0.5)	DMAc	18	90	34
5	NBS (0.5)	toluene	18	25	68
6	NBS (0.5)	toluene	24	25	69
7	NBS (0.1)	toluene	18	25	30
8	NBS (0.1)	toluene	18	80	47
9	NBS (1)	toluene	18	25	40
10	NBS (0.3)	toluene	12	25	76
11	NBS/K_2_CO_3_ (0.3:1)	toluene	18	25	nr^d^
12	NBS/4 Å MS (0.3:2)	toluene	12	25	15
13	LiBr (0.3)	toluene	12	25	nr
14	CuBr	toluene	12	25	42

^a^Reaction conditions: styrene (0.5 mmol), phenylboronic acid (0.5 mmol), Pd(OAc)_2_ (5 mol %), additive and dry solvent (1 mL) for 12 h at 25 °C. ^b^Isolated yield. ^c^Product was characterized by GC–MS, ^1^H and ^13^C NMR. ^d^Reaction was stirred under air.

The optimized Heck cross-coupling conditions were employed to examine the arylation of substituted olefins and phenylboronic acid. The results are presented in Table 4. As shown in Table 4, this coupling procedure tolerates various functional groups to afford the desired product (**3**). The compatibility of halo-substituted styrenes is synthetically useful since the products could be easily modified further to form synthetic building blocks. Thus, the coupling of 4-fluorostyrene (**2q**), 4-bromostyrene (**2b**) and 4-chlorostyrene (**2r**) through oxidative Heck reaction led to the corresponding products in 65–69% yields, respectively (Table 4, entries 2–4). Furthermore, the electron-withdrawing groups on styrene, such as 3-nitrostilbene (**2d**) and 4-trifluorostilbene (**2k**) resulted in the formation **3e** and **3k** in 76% and 70% yields, respectively (Table 4, entries 5 and 6). However, the electron-donating substituent on olefin lessened the reaction rate and thus led to poor yield of product **3f** (Table 4, entry 7). The reaction with aliphatic alkenes, such as *tert*-butyl acrylate (**2n**) or ethyl acrylate (**2o**), allyl acetate (**2s**) and *n*-heptene (**2t**) afforded the corresponding coupled products in moderate yields, respectively (Table 4, entries 9–12).

**Table 4:** Substrate scope in the Heck arylation reaction of phenylboronic acids with olefins.^a^

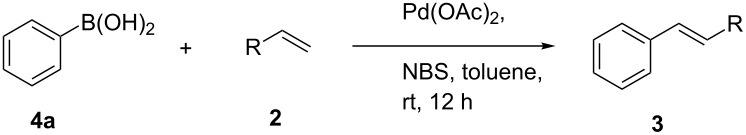			

Entry	Substrate (**2**)	Product (**3**)^b^	Yield (%)^c^

1	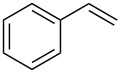 **2a**	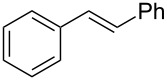 **3a**	76
2	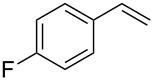 **2q**	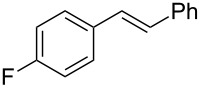 **3r**	65^d^
3	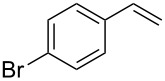 **2b**	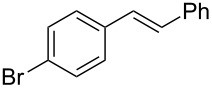 **3b**	69
4	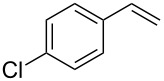 **2r**	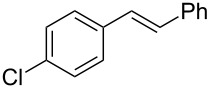 **3s**	66
5	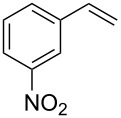 **2d**	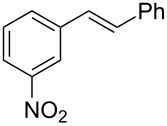 **3e**	76
6	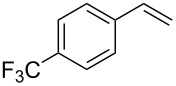 **2k**	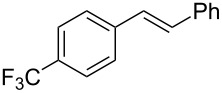 **3k**	70
7	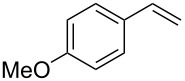 **2e**	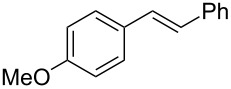 **3f**	30
8	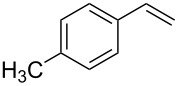 **2c**	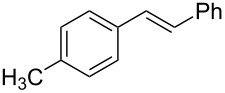 **3c**	70
9	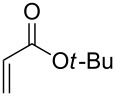 **2n**	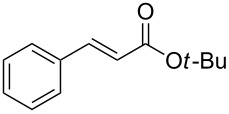 **3o**	50
10	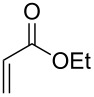 **2o**	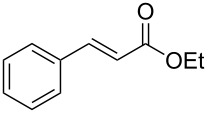 **3p**	42
11	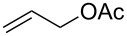 **2s**	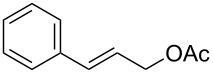 **3t**	38
12	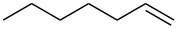 **2t**	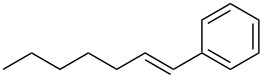 **3u**	44^e^

^a^Reaction conditions: styrene (1.0 mmol), phenylboronic acid (1.0 mmol), catalyst (5 mol %), *N*-bromosuccinimide (30 mol %), and toluene (2 mL) under nitrogen for 12 h. ^b^Product was characterized by GC–MS, ^1^H and ^13^C NMR. ^c^Isolated yield. ^d^Determined by GC–MS. ^e^*E*/*Z* ratio 20:1 by ^1^H NMR, terminal/internal 4/1.

To expand the scope of this cross-coupling, these conditions were then applied to a variety of boronic acids and styrene (Table 5). For a diverse set of boronic acids, cross-coupling proceeded smoothly with **2a** in moderate to good yields. In this case, the procedure also tolerated a range of functional groups, such as bromo, chloro, nitro, methoxy, and alkyl groups. The arylboronic acids with electron-withdrawing substituents furnished good yields of coupled product as compared to the electron-donating substituents. For example, 4-nitro (**4e**) and 3-nitrophenylboronic acid (**4f**) were reacted smoothly with styrene to afford the corresponding products in 75% and 73% yields, respectively (Table 5, entries 5 and 6).

**Table 5:** Substrate scope in Heck arylation reaction of phenylboronic acids with olefins.^a^

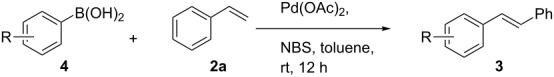			

Entry	Substrate	Product^b^	Yield (%)^c^

1	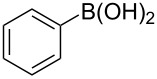 **4a**	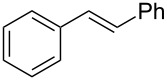 **3a**	76
2	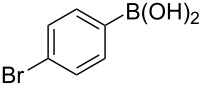 **4b**	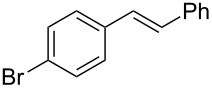 **3b**	69
3	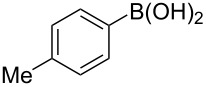 **4c**	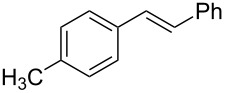 **3c**	67
4	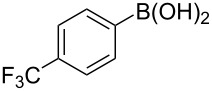 **4d**	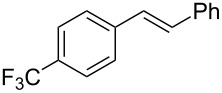 **3k**	72
5	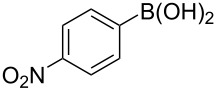 **4e**	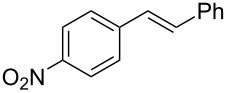 **3v**	75
6	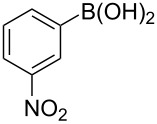 **4f**	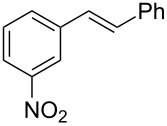 **3e**	73
7	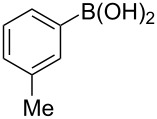 **4g**	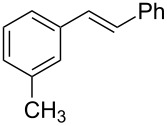 **3l**	60
8	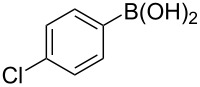 **4h**	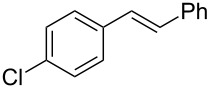 **3s**	62
9	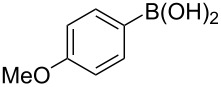 **4i**	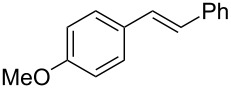 **3f**	40

^a^Reaction conditions: similar to Table 4. ^b^Product was characterized by GC–MS, ^1^H and ^13^C NMR. ^c^Isolated yield.

## Conclusion

In summary, we have developed two new protocols for oxidative Heck reactions employing Pd(OAc)_2_ as a catalytic precursor. The first method is based on coupling between various olefins and aryl halides utilizing an imidazole-based secondary phosphine oxide ligated palladium complex (**6**) under mild conditions. The yields of products obtained were excellent and in high regioselectivity. Compared with the previously described procedures for the Heck reaction of aryl halides as substrates employing a SPO–Pd complex as a catalyst, the method reported here has the advantages of having a stable catalyst system, general substrate scope, and mild reaction conditions (60 °C). Secondly, we also developed the Heck reaction of arylboronic acids with various alkenes employing *N*-bromosuccinimide as an additive and catalyzed by Pd(OAc)_2_, under base- and ligand-free conditions at 25 °C. The yields of the coupled products are moderate to good.

## Supporting Information

File 1 General procedure for Heck reactions, preparation of complex **6** and characterization data.

## References

[1] Douney A M, Overman L E (2003). Chem Rev.

[2] Nicolaou K C, Bulger P G, Sarlah D (2005). Angew Chem, Int Ed.

[3] Jang M, Cai L, Udeani G O, Slowing K V, Thomas C F, Beecher C W W, Fong H H S, Farnsworth N R, Kinghorn A D, Mehta R G (1997). Science.

[4] Elmali N, Baysal O, Harma A, Esenkaya I, Mizrak B (2007). Inflammation.

[5] Karuppagounder S S, Pinto J T, Xu H, Chen L-H, Beal M F, Gibson G E (2009). Neurochem Int.

[6] Gurusamy N, Lekli I, Mukherjee S, Ray D, Ahsan M K, Gherghiceanu M, Popescu L M, Das D K (2010). Cardiovasc Res.

[7] Tsuji J (1995). Palladium Reagents and Catalysts: Innovations in Organic Synthesis.

[8] Bräse S, de Meijere A, Diederich F, Stang P J (1998). Cross-Coupling of Organyl Halides with Alkenes: the Heck Reaction. Metal-Catalyzed Cross-Coupling Reactions.

[9] Sehnal P, Taylor R J K, Fairlamb I J S (2010). Chem Rev.

[10] Mizoroki T, Mori K, Ozaki A (1971). Bull Chem Soc Jpn.

[11] Heck R F, Nolley J P (1972). J Org Chem.

[12] Crisp G T (1998). Chem Soc Rev.

[13] Link J T, Overman L E, Diedrich F, Stang P J (1998). Intramolecular Heck Reactions in Natural Product Chemistry. Metal-Catalyzed Cross-Coupling Reactions.

[14] Beletskaya I, Cheprakov A V (2000). Chem Rev.

[15] Liu L-j, Wang F, Wang W, Zhao M-x, Shi M (2011). Beilstein J Org Chem.

[16] Grasa G A, Singh R, Stevens E D, Nolan S P (2003). J Organomet Chem.

[17] Ozawa F, Kubo A, Hayashi T (1992). Chem Lett.

[18] Littke A F, Fu G C (1999). J Org Chem.

[19] Stambuli J P, Stauffer S R, Shaughnessy K H, Hartwig J F (2001). J Am Chem Soc.

[20] Hansen A L, Ebran J-P, Ahlquist M, Norrby P-O, Skrydstrup T (2006). Angew Chem, Int Ed.

[21] Fleckenstein C A, Plenio H (2010). Chem Soc Rev.

[22] Parshall G W, Ittel S D (1992). Homogeneous Catalysis.

[23] Albéniz A C, Carrera N (2011). Eur J Inorg Chem.

[24] Li G Y (2001). Angew Chem, Int Ed.

[25] Jiang X-b, Minnaard A J, Feringa B L, de Vries J G (2004). J Org Chem.

[26] Ackermann L, Born R (2005). Angew Chem, Int Ed.

[27] Xu H, Ekoue-Kovi K, Wolf C (2008). J Org Chem.

[28] Ackermann L, Potukuchi H K, Kapdi A R, Schulzke C (2010). Chem–Eur J.

[29] Li G Y, Zheng G, Noonan A F (2001). J Org Chem.

[30] Wolf C, Lerebours R (2003). J Org Chem.

[31] Punji B, Mague J T, Balakrishna M S (2007). Inorg Chem.

[32] Wei C-H, Wu C-E, Huang Y-L, Kultyshev R G, Hong F-E (2007). Chem–Eur J.

[33] Dieck H A, Heck R F (1975). J Org Chem.

[34] Hayashi T, Yamasaki K (2003). Chem Rev.

[35] Itoh T, Mase T, Nishikata T, Iyama T, Tachikawa H, Kobayashi Y, Yamamoto Y, Miyaura N (2006). Tetrahedron.

[36] Vandyck K, Mattys B, Willen M, Robeyns K, Van Meervelt L, Van der Eycken J (2006). Org Lett.

[37] Bazin M-A, El Kihel L, Lancelot J-C, Rault S (2007). Tetrahedron Lett.

[38] Motokura K, Hashimoto N, Hara T, Mitsudome T, Mizugaki T, Jitsukawa K, Kaneda K (2011). Green Chem.

[39] Yoo K S, Park C P, Yoon C H, Sakaguchi S, O’Neill J, Jung K W (2007). Org Lett.

[40] Andappan M M S, Nilsson P, Larhed M (2004). Chem Commun.

[41] Lindh J, Sävmarker J, Nilsson P, Sjöberg P J R, Larhed M (2009). Chem–Eur J.

[42] Odell L R, Lindh J, Gustafsson T, Larhed M (2010). Eur J Org Chem.

[43] Nordqvist A, Björkelid C, Andaloussi M, Jansson A M, Mowbray S L, Karlén A, Larhed M (2011). J Org Chem.

[44] Likhar P R, Roy M, Roy S, Subhas M S, Kantam M L, Sreedhar B (2008). Adv Synth Catal.

[45] Delcamp J H, Brucks A P, White M C (2008). J Am Chem Soc.

[46] Leng Y, Yang F, Wei K, Wu Y (2010). Tetrahedron.

[47] Sakaguchi S, Yoo K S, O’Neill J, Lee J H, Stewart T, Jung K W (2008). Angew Chem, Int Ed.

[48] Ruan J, Li X, Saidi O, Xiao J (2008). J Am Chem Soc.

[49] Gottumukkala A L, Teichert J F, Heijnen D, Eisink N, Van Dijk S, Ferrer C, van den Hoogenband A, Minnaard A J (2011). J Org Chem.

[50] Li T, Qu X, Zhu Y, Sun P, Yang H, Shan Y, Zhang H, Liu D, Zhang X, Mao J (2011). Adv Synth Catal.

[51] Werner E W, Sigman M S (2010). J Am Chem Soc.

[52] Sun P, Zhu Y, Yang H, Yan H, Lu L, Zhang X, Mao J (2012). Org Biomol Chem.

[53] Mino T, Koizumi T, Suzuki S, Hirai K, Kajiwara K, Sakamoto M, Fujita T (2012). Eur J Org Chem.

[54] Dubrovina N V, Borner A (2004). Angew Chem, Int Ed.

[55] Ackermann L (2010). Isr J Chem.

[56] Shaikh T M, Weng C-M, Hong F-E (2012). Coord Chem Rev.

[57] Ackermann L, Kapdi A R, Schulzke C (2010). Org Lett.

[58] Hu D-F, Weng C-M, Hong F-E (2011). Organometallics.

[59] Zhang Y, Xing H, Xie W, Wan X, Lai Y, Ma D (2013). Adv Synth Catal.

