# Supplementary material for: Palladium(II)-catalyzed Heck reaction of aryl halides and arylboronic acids with olefins under mild conditions
Source: Beilstein J Org Chem. 2013 Aug 5;9:1578–88. doi: 10.3762/bjoc.9.180 (PMC3740572; doi:10.3762/bjoc.9.180)
Supplement: File 1 — General procedure for Heck reactions, preparation of complex 6 and characterization data. [file Beilstein_J_Org_Chem-09-1578-s001.pdf]

Supporting Information

for

Palladium(II)-catalyzed Heck reaction of aryl halides and arylboronic acids with olefins under mild conditions

Tanveer Mahamadali Shaikh and Fung-E Hong*

Address: Department of Chemistry, National Chung Hsing University, 250 Kuo-Kuang Road, Taichung, Taiwan (R.O.C.)

Email: Fung-E Hong - fehong@dragon.nchu.edu.tw

* Corresponding author

General procedure for Heck reactions, preparation of complex 6 and characterization data

Table of Contents

1. General information
2. Preparation of 1-(2-methoxyphenyl)-1*H*-imidazole **5** and its complex with palladium (**6**)
3. Typical procedure Table 2: Heck reaction of aryl halides with olefins
4. Typical procedure (Table 4 and 5): Heck reaction of phenylboronic acids with olefins
5. Spectral details

1. General information

All reactions were carried out under a nitrogen atmosphere using standard Schlenk techniques. Commercially available solvents were used. All processes of separations of the products were performed by column chromatography. GC–MS analysis was performed on an Agilent 5890 gas chromatograph (Restek Rtx-5MS fused silica capillary column: 30 m, 0.25 mm, 0.5 μm) with an Agilent 5972 mass selective detector. Routine ^{1}H NMR spectra were recorded on a Varian-400 spectrometer at 400.00 MHz. The chemical shifts are reported in ppm relative to internal standard TMS (δ = 0.0). ^{13}C NMR spectra were recorded at 100.7 MHz, respectively.

2. Preparation of 1-(2-methoxyphenyl)-1*H*-imidazole **5** and its complex with palladium (**6**) [1]

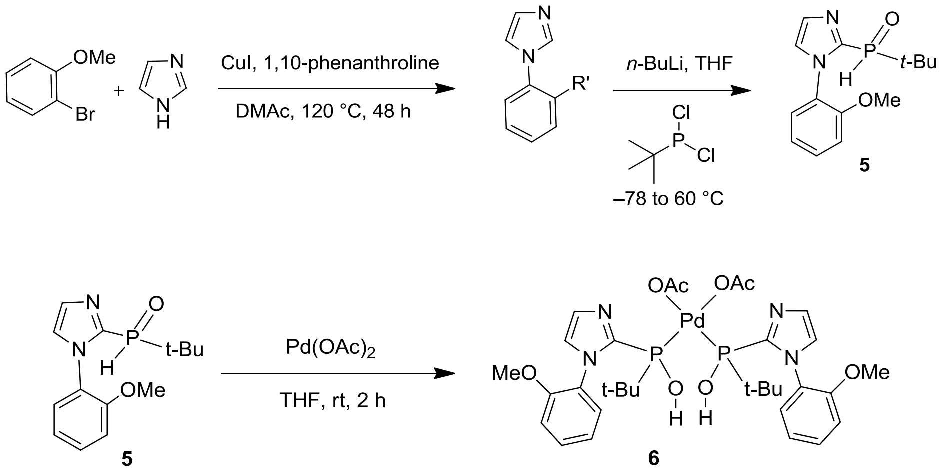

To a mixture of CuI (1.14 g, 6.0 mmol), 1,10-phenanthroline (1.08 g, 6.0 mmol), K_3PO_4 (19.10 g, 90.0 mmol) and imidazole (2.86 g, 42.0 mmol) was added *N*,*N*-dimethylformamide (50 mL) at 25 °C. The resulting mixture was stirred for 20 min at

25 °C, and then 2-bromoanisole (3.74 mL, 30 mmol) was added dropwise. The solution was then heated under stirring at 120 °C for 48 h. After completion of the reaction, the reaction mixture was cooled and diluted with ethyl acetate (20 mL). This solution was then filtered through a pad of silica gel, and washed with ethyl acetate (3 × 20 mL). The organic layer was washed with brine (40 × 2 mL) and dried over $MgSO_4$, concentrated using a rotary evaporator. The residue was purified by flash chromatography (hexane/ethyl acetate 3:1) to afford the corresponding Ullmann adduct in 78% yield (3.37 g, 23.40 mmol).

Then the solution of the Ullmann adduct (1.0 mmol, 0.144 g) in 10 mL of anhydrous THF was cooled to −78 °C. To this mixture, 2.5 M *n*-BuLi in *n*-hexane (1.1 mmol, 0.44 mL) was added very slowly. The suspension was then warmed to 0 °C and stirred for another 30 min. The whole reaction mixture was subsequently transferred to the flask, which contained *tert*-butyldichlorophosphine (1.0 mmol, 0.159 g) in 10 mL of anhydrous THF at 0 °C. The reaction mixture was stirred to 15 min and then heated gradually to 60 °C for another 2 h. After completion the reaction was quenched with ethyl acetate (5 mL) followed by 1.0 M NH_4Cl solution (10 mL) at 0 °C. After being stirred for a few minutes, the organic layer was separated and the aqueous layer was extracted twice with ethyl acetate (15 × 2 mL). The combined organic layer was dried over $MgSO_4$, and concentrated using a rotary evaporator. The residue was purified by flash chromatography (ethyl acetate/methanol 20:1) to afford a white solid (**5**) in 45% yield (0.45 mmol, 111.0 mg). The procedures for the preparation of **6** can be found in the literature [1].

Selected spectroscopic data for **5**:

^{1}H NMR ($CDCl_3$, δ/ppm) 1.15, 1.11 (d, 9H, *t*-Bu, $^{3}J_{P\text{-}CC3H9}$ = 16 Hz), 3.82 (s, 3H, OMe), 7.08-7.03 (m, 2H, Ar), 7.35, 7.19 (s, 2H, C=C), 7.47-7.43 (m, 2H, Ar), 7.55, 6.36 (d, 1H, P-H, $^{1}J_{P\text{-}H}$ = 476 Hz); ^{13}C NMR ($CDCl_3$, δ/ppm) 153.8, 140.5, 139.2, 130.7, 130.2, 130.0, 128.6, 125.6, 120.5, 111.6, 55.6, 32.74, 32.0, 23.65; ^{31}P NMR ($CDCl_3$, δ/ppm) 29.5 (d, P-H, $^{1}J_{P\text{-}H}$ = 476 Hz); MS (EI, *m*/*z*) 278.1 $[M]^+$.

3. Typical procedure Table 2: Heck reaction of aryl halides with olefins

A Schlenk tube was charged with readily prepared complex **6** (2 mol %) under a nitrogen atmosphere. Then styrene (104 mg, 1 mmol), K_2CO_3 (276 mg, 2 mmol) and dimethyl formamide (1 mL) were added at 25 °C. The reaction mixture was stirred under a nitrogen atmosphere. To this solution, bromobenzene (0.157 mg, 1 mmol) was then added with a syringe and the reaction mixture was heated at 60 °C in an oil bath for 12 h. After completion of the reaction, the reaction mixture was cooled to room temperature, diluted with EtOAc, and filtered through a pad of Celite. The collected filtrate was then concentrated using a rotary evaporator, and the residue was purified by flash chromatography (10% EtOAc/hexane), which yielded 172 mg (96%) of *trans*-stilbene.

4. Typical procedure (Table 4 and 5): Heck reaction of phenylboronic acids with olefins

A Schlenk tube was charged with palladium acetate (5 mol %, 0.005 g), *N*-bromosuccinimide (30 mol % 0.026 g) and phenylboronic acid (0.5 mmol). This was

subjected to vacuum for 10 min and then toluene (1 mL) was added. To this mixture styrene (0.5 mmol in 0.5 mL of toluene) was added dropwise with a syringe. The resulting suspension was stirred at ambient temperature for 12 h. The reaction was monitored by TLC. After completion of the reaction, the reaction was diluted with EtOAc and filtered through a pad of Celite. The organic layer was then extracted with ethylacetate (10 × 3 mL) and washed with water. The organic layer was collected separately and dried over anhydrous Na_2SO_4 and concentrated under reduced pressure to obtain crude residue. The product was purified by flash chromatography (5% EtOAc/hexane), which yielded 68 mg (76%) of *trans*-stilbene.

5. Spectral details

***trans*-Stilbene (3a)**

^{1}H NMR (400 MHz, $CDCl_3$) δ ppm 7.11 (s, 2H), 7.27 (t, *J* = 8.0 Hz, 2H), 7.35 (t, *J* = 8.0 Hz, 4H), 7.51 (d, *J* = 8.0 Hz, 4H); ^{13}C NMR (101 MHz, $CDCl_3$) δ ppm 126.4, 127.5, 128.6, 137.2.

(*E*)-1-Bromo-4-styrylbenzene (3b) [2]

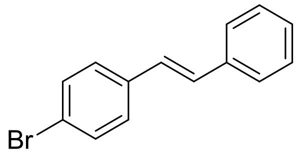

^{1}H NMR (400 MHz, $CDCl_3$) δ ppm 7.05 (1H, d, *J* = 16.0 Hz), 7.10 (1H, d, *J* = 16.0 Hz), 7.23-7.37 (5H, m), 7.44-7.50 (4H, m). ^{13}C NMR (101 MHz, $CDCl_3$) δ ppm 121.2, 126.5, 127.3, 127.8, 127.9, 128.6, 129.3, 131.7, 136.2, 136.8.

(*E*)-1-Methyl-4-styrylbenzene (3c) [2]

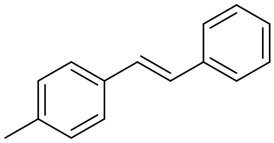

^{1}H NMR (400 MHz, $CDCl_3$) δ ppm 2.11 (3H, s), 6.85 (1H, d, *J* = 16.0 Hz), 6.91 (1H, d, *J* = 16.0 Hz), 7.04 (1H, d, *J* = 8.0 Hz), 7.09-7.24 (7H, m), 7.62 (1H, d, *J* = 8.0 Hz). ^{13}C NMR (101 MHz, $CDCl_3$) δ ppm 21.0, 126.3, 126.4, 127.2, 127.5, 128.0, 128.1, 128.4, 128.5, 128.7, 129.2, 134.3, 137.2, 137.3.

(*E*)-2-Styrylnaphthalene (3d)

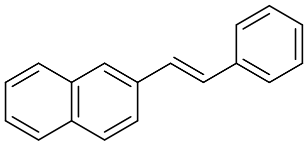

^{1}H NMR (400 MHz, $CDCl_3$) δ ppm 7.18-7.28 (3H, m), 7.34-7.47 (4H, m), 7.54 (2H, d, *J* = 8.1 Hz), 7.71(1H, d, *J* = 8.1 Hz), 7.78-7.82 (4H, m). ^{13}C NMR (100 MHz, $CDCl_3$) δ ppm 123.4, 125.8, 126.2, 126.4, 126.6, 127.6, 127.9, 128.2, 128.6, 128.9, 132.9, 133.6, 134.7, 137.2.

(*E*)-1-Nitro-3-styrylbenzene (3e)

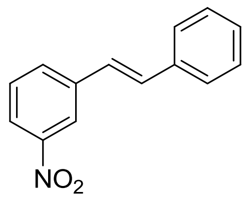

^{1}H NMR (400 MHz, $CDCl_3$) δ ppm 7.13 (1H, d, *J* = 16.0 Hz), 7.21-7.41 (5H, m), 7.50-7.55 (3H, m), 7.79 (1H, d, *J* = 8.0 Hz), 8.37 (1H, d, *J* = 4.0 Hz). ^{13}C NMR (101 MHz, $CDCl_3$) δ ppm 120.7, 121.9, 126.0, 126.7, 128.4, 128.7, 129.4, 131.6, 132.1, 136.1, 139.0, 148.6.

(*E*)-1-Methoxy-4-styrylbenzene (3f) [2]

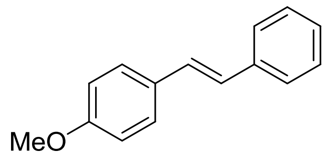

^{1}H NMR (400 MHz, $CDCl_3$) δ ppm 3.80 (3H, s), 6.87 (2H, d, *J* = 8.5 Hz), 6.89 (1H, d, *J* = 16.0 Hz), 7.04 (1H, d, *J* = 16.0 Hz), 7.22 (1H, t, *J* = 8.0 Hz), 7.34 (1H, t, *J* = 8.0 Hz), 7.43-7.49 (5H, m). ^{13}C NMR (100 MHz, $CDCl_3$) δ ppm 55.2, 114.0, 126.2, 126.5, 127.1, 127.6, 128.1, 128.5, 130.0, 137.5, 159.2.

(*E*)-4-Styrylbenzaldehyde (3h)

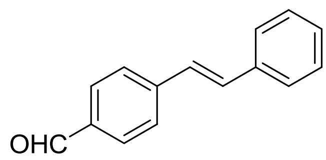

^{1}H NMR (400 MHz, $CDCl_3$) δ ppm 7.12 (1H, d, *J* = 16.0 Hz), 7.22-7.40 (4H, m), 7.54 (2H, d, *J* = 2.0 Hz), 7.64 (2H, d, *J* = 2.0 Hz), 7.85 (2H, d, *J* = 12.0 Hz), 9.98 (1H, s). ^{13}C NMR (100 MHz, $CDCl_3$) δ ppm 126.8, 127.2, 128.4, 128.7, 130.1, 132.1, 135.2, 136.4 143.3, 191.5.

(*E*)-1-(4-Styrylphenyl)ethanone (3i) [2]

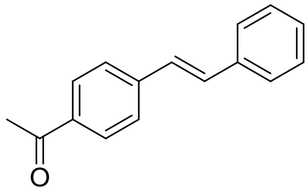

^{1}H NMR (400 MHz, $CDCl_3$) δ ppm 2.60 (3H, s), 7.14 (1H, d, *J* = 16.0 Hz), 7.20 (1H, d, *J* = 16.0 Hz), 7.38-7.58 (7H, m) 7.96 (2H, d, *J* = 12.0 Hz). ^{13}C NMR (100 MHz, $CDCl_3$) δ ppm 26.3, 126.2, 126.6, 127.1, 128.1, 128.6, 131.2, 135.6, 136.4, 141.7, 197.2.

(*E*)-4-Styrylbenzonitrile (3j) [3]

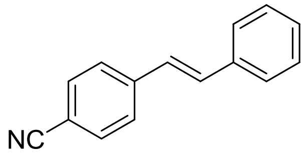

^{1}H NMR (400 MHz, $CDCl_3$) δ ppm 7.04 (1H, d, *J* = 16.0 Hz), 7.17 (1H, d, *J* = 16.0 Hz), 7.30-7.38 (3H, m), 7.49-7.74 (6H, m). ^{13}C NMR (100 MHz, $CDCl_3$) δ ppm 110.3, 118.8, 126.2, 126.5, 126.6, 126.7, 127.7, 128.2, 128.4, 128.6, 132.1, 132.2, 132.6, 136.0, 141.6, 143.2.

(*E*)-1-Styryl-4-(trifluoromethyl)benzene (3k)

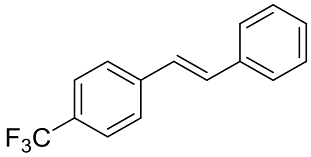

^{1}H NMR (400 MHz, $CDCl_3$) δ ppm 7.15 (1H, d, *J* = 16.0 Hz), 7.24 (1H, d, *J* = 16.0 Hz), 7.36-7.45 (3H, m), 7.57-7.66 (6H, m). ^{13}C NMR (100 MHz, $CDCl_3$) δ ppm 125.5, 126.5, 126.7, 127.0, 128.2, 128.7, 131.1, 136.5, 140.7.

(*E*)-1-Methyl-3-styrylbenzene (3l)

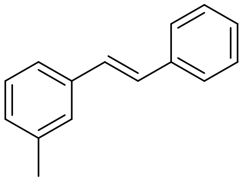

^{1}H NMR (400 MHz, $CDCl_3$) δ ppm 2.20 (3H, s), 6.92 (3H, d, *J* = 4.0 Hz), 7.08-7.20 (6H, m), 7.33 (2H, d, *J* = 8.0 Hz). ^{13}C NMR (100 MHz, $CDCl_3$) δ ppm 21.3, 123.6, 126.3, 127.1, 127.3, 128.3, 128.4, 128.5, 128.6, 137.1, 137.2, 137.9.

(*E*)-1,3-Dimethyl-5-styrylbenzene (3m)

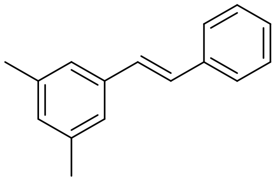

^{1}H NMR (400 MHz, $CDCl_3$) δ ppm 2.32 (6H, s), 6.90 (1H, s), 7.05 (2H, d, *J* = 4.4 Hz), 7.13 (2H, s), 7.21-7.29 (m, 1H), 7.32-7.35 (2H, m), 7.48-7.50 (2H, m). ^{13}C NMR

(100 MHz, $CDCl_3$) δ ppm 21.2, 124.3, 126.3, 127.3, 128.2, 128.5, 128.8, 129.3, 137.1, 137.4, 138.0.

(*E*)-2-Styrylpyridine (3n) [2]

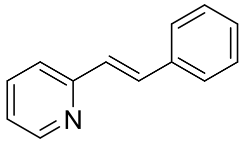

^{1}H NMR (400 MHz, $CDCl_3$) δ ppm 7.11-7.18 (2H, m), 7.25-7.38 (4H, m), 7.56-7.66 (4H, m), 8.59 (1H, d, *J* = 4.0 Hz). ^{13}C NMR (100 MHz, $CDCl_3$) δ ppm 121.9, 122.0, 127.0, 127.8, 128.2, 128.6, 132.6, 136.4, 136.5, 149.5, 155.5.

(*E*)-*tert*-Butyl cinnamate (3o) [4]

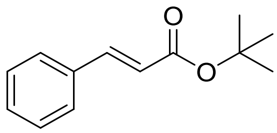

^{1}H NMR (400 MHz, $CDCl_3$) δ ppm 1.54 (9H, s), 6.37 (1H, d, *J* = 16.0 Hz), 7.35-7.37 (3H, m), 7.49 (2H, dd, *J* = 4.0 Hz), 7.59 (1H, d, *J* = 16.0 Hz). ^{13}C NMR (100 MHz, $CDCl_3$) δ ppm 28.1, 80.3, 120.0, 127.8, 128.7, 129.8, 134.5, 143.4, 166.2.

(*E*)-Ethyl cinnamate (3p) [4]

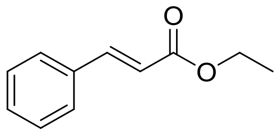

^{1}H NMR (400 MHz, $CDCl_3$) δ ppm 1.27-1.32 (3H, m), 4.20-4.26 (2H, m), 6.42 (1H, d, *J* = 16.0 Hz), 7.31-7.48 (5H, m), 7.66 (1H, d, *J* = 16.0 Hz). ^{13}C NMR (100 MHz, $CDCl_3$) δ ppm 14.0, 60.1, 118.0, 127.7, 128.5, 129.9, 134.1, 144.2, 166.6.

(*E*)-Methyl cinnamate (3q) [4]

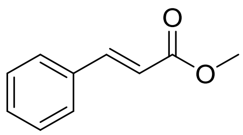

^{1}H NMR (400 MHz, $CDCl_3$) δ ppm 3.67 (3H, s), 6.34 (1H, d, *J* = 16.0 Hz), 7.23-7.39 (5H, m), 7.59 (1H, d, *J* = 16.0 Hz). ^{13}C NMR (100 MHz, $CDCl_3$) δ ppm 51.1, 117.3, 127.6, 128.4, 129.8, 133.9, 144.3, 166.8.

References

[1] Hu, D.-F.; Weng, C.-M.; Hong, F.-E. *Organometallics*, **2011**, *30*, 1139–1147.

[2] a) Sore, H. F.; Boehner, C. M.; MacDonald, S. J. F.; Norton, D.; Fox, D. J.; Spring, D. R. *Org. Biomol. Chem*. **2009**, *7*, 1068–1071; b) Cui, X.; Li, Z.; Tao, C.-Z.; Xu, Y.; Li, J.; Liu L.; Guo, Q.-X. *Org. Lett.* **2006**, *8*, 2467–2470.

[3] Iranpoor, N.; Firouzabadi, H.; Azadi, R. *Eur. J. Org. Chem*. **2007**, 2197–2201.

[4] Sun, P.; Qu, X.; Li, T.; Zhu, Y.; Yang, H.; Xing, Z.; Mao, J. *Synlett*. **2012**, 150–154.
